# MALT1-dependent cleavage of CYLD promotes NF-κB signaling and growth of aggressive B-cell receptor-dependent lymphomas

**DOI:** 10.1038/s41408-023-00809-7

**Published:** 2023-03-15

**Authors:** Marthe Minderman, Hildo C. Lantermans, Leonie J. Grüneberg, Saskia A. G. M. Cillessen, Richard J. Bende, Carel J. M. van Noesel, Marie José Kersten, Steven T. Pals, Marcel Spaargaren

**Affiliations:** 1 Department of Pathology, Amsterdam UMC, location University of Amsterdam, Amsterdam, The Netherlands; 2 Lymphoma and Myeloma Center Amsterdam (LYMMCARE), Amsterdam, The Netherlands; 3 Cancer Center Amsterdam (CCA), Cancer Biology and Immunology, Target & Therapy Discovery, Amsterdam, The Netherlands; 4 Department of Pathology, Amsterdam UMC, location VU University, Amsterdam, Netherlands; 5 Department of Hematology, Amsterdam UMC, location University of Amsterdam, Amsterdam, The Netherlands

**Keywords:** B-cell lymphoma, Cell signalling

## Abstract

The paracaspase mucosa-associated lymphoid tissue 1 (MALT1) is a protease and scaffold protein essential in propagating B-cell receptor (BCR) signaling to NF-κB. The deubiquitinating enzyme cylindromatosis (CYLD) is a recently discovered MALT1 target that can negatively regulate NF-κB activation. Here, we show that low expression of CYLD is associated with inferior prognosis of diffuse large B-cell lymphoma (DLBCL) and mantle cell lymphoma (MCL) patients, and that chronic BCR signaling propagates MALT1-mediated cleavage and, consequently, inactivation and rapid proteasomal degradation of CYLD. Ectopic overexpression of WT CYLD or a MALT1-cleavage resistant mutant of CYLD reduced phosphorylation of IκBα, repressed transcription of canonical NF-κB target genes and impaired growth of BCR-dependent lymphoma cell lines. Furthermore, silencing of CYLD expression rendered BCR-dependent lymphoma cell lines less sensitive to inhibition of NF-κΒ signaling and cell proliferation by BCR pathway inhibitors, e.g., the BTK inhibitor ibrutinib, indicating that these effects are partially mediated by CYLD. Taken together, our findings identify an important role for MALT1-mediated CYLD cleavage in BCR signaling, NF-κB activation and cell proliferation, which provides novel insights into the underlying molecular mechanisms and clinical potential of inhibitors of MALT1 and ubiquitination enzymes as promising therapeutics for DLBCL, MCL and potentially other B-cell malignancies.

## Introduction

Diffuse large B-cell lymphoma (DLBCL) and mantle cell lymphoma (MCL) are aggressive subtypes of B-cell non-Hodgkin lymphoma (B-NHL) characterized by a poor prognosis. The NF-κB signaling pathway is constitutively active in activated B-cell-like (ABC) DLBCL as a result of oncogenic mutations in the Toll-like receptor (TLR) signaling pathway and the B-cell antigen receptor (BCR) signaling pathway [1–3]. Although these mutations are rare in MCL, a subset of MCL cell lines was also demonstrated to be dependent on BCR-mediated, chronic activation of canonical NF-κB signaling [4, 5]. Intriguingly, analysis of the immunoglobulin heavy chain (*IGHV*) gene repertoire points toward a possible role for chronic (super) antigen-dependent BCR activation in a subset of ABC DLBCL and MCL patients [6, 7]. Promising single-agent efficacy of the Bruton’s tyrosine kinase (BTK) inhibitor ibrutinib supports the notion that BCR signaling is essential in the pathogenesis of ABC DLBCL and MCL [8, 9].

Formation of the caspase recruitment domain family member 11 (CARD11)—B-cell lymphoma 10 (BCL10)—mucosa-associated lymphoid tissue lymphoma translocation gene 1 (MALT1) complex (CBM complex) is a key event in linking BCR antigen recognition to canonical NF-κB activation (reviewed by Thome et al. [10]). MALT1 functions as a scaffolding protein allowing recruitment and activation of the E3-ubiquitin ligase tumor necrosis factor receptor (TNFR)-associated factor 6 (TRAF6). TRAF6 mediates Lys-63-linked polyubiquitination of various targets including the regulatory gamma subunit of the inhibitor of IκB kinase (IKK) complex (IKK-γ or NEMO), allowing full activation of the IKK complex [11, 12]. Next to its function as scaffold protein, MALT1 possesses protease activity and is able to cleave and inactivate negative regulators of NF-κB signaling, e.g., RelB and A20 [13–15]. Inhibition of MALT1 proteolytic activity impairs survival in a subset of MCL and ABC DLBCL cell lines as well as in ex vivo cultured primary DLBCL, suggesting that cleavage of these substrates is essential for lymphomagenesis [16–22].

Another negative regulator of NF-κB is the deubiquitinating enzyme cylindromatosis (CYLD), which can hydrolyze Lys-63-linked ubiquitin chains of various targets, including TRAF2, TRAF6 and IKK-γ [23–25]. Whereas deletion or mutation of the gene encoding CYLD is a frequently occurring genomic aberration in multiple myeloma (MM), these events are extremely uncommon in DBLCL and MCL [26, 27]. Previous studies have indicated that CYLD deubiquitinase activity can be repressed by IKK-mediated phosphorylation as well as by (para)caspase-mediated cleavage, including, at least in T-cells, by MALT1 [28–31]. This prompted us to investigate if in DBLCL and MCL similar, non-genetic, mechanisms contribute to inactivation of CYLD. Here, we demonstrate that low expression of CYLD is associated with a poor prognosis of (ABC) DLBCL and MCL patients, and that chronic BCR signaling controls cleavage-mediated inactivation of CYLD by the paracaspase MALT1, followed by rapid proteasomal degradation. Ectopic overexpression of WT CYLD or a MALT1-cleavage resistant mutant of CYLD reduced NF-κB activity and growth of BCR-dependent lymphoma cell lines. Furthermore, silencing of CYLD renders BCR-dependent cell lines less sensitive to BCR signalosome inhibitors, indicating that their inhibitory effects on cell proliferation and NF-κΒ activity are partially CYLD dependent.

## Materials and methods

### Cell culture and primary cell isolation

DLBCL and MCL cell lines were cultured as previously described [32]. Primary DLBCL and peripheral blood derived MCL cells were obtained after routine diagnostics or follow-up procedures at the Amsterdam University Medical Centers, the Netherlands. DLBCLs were purified using Ficoll and B cell isolation kit (Miltenyi Biotec, Bergisch Gladbach, Germany). MCLs were sorted on a BD-FACS-Aria IIu to obtain CD5+/CD19 + cells. DLBCLs were classified as either GCB- or non-GCB like, using the immunohistochemical algorithm of Hans et al. [33]. This study was approved by the AMC Medical Committee on Human Experimentation. Informed consent was obtained in accordance with the revised Declaration of Helsinki 2008.

### Cell viability and cell cycle analyses

For growth assays, 10–50 × 10^3^ cells were plated in 96-well plates and treated as indicated. Cells were analyzed on a FACSCanto II flow cytometer (BD Biosciences, Franklin Lakes, New Jersey, USA) and viability was determined using 7-AAD viability staining solution (Thermo Fisher Scientific, Waltham, Massachusetts, USA). For long-term competition assays, cells were passaged every 3–4 days at a density of 0.3 × 10^6^ cells/ml. For cell cycle analysis, cells were incubated for 1 h with 20 μM BrdU (Sigma Aldrich, Saint Louis, Missouri, USA) and subsequently stained with anti-BrdU FITC (clone B44, BD Biosciences) and 100 nM To-pro 3 Iodide (Invitrogen Life Technologies, Carlsbad, California, USA).

### Statistical analysis

Survival analysis was performed using the Kaplan–Meier method and log-rank test. Data are presented as mean ± SD of at least three independently performed experiments. Experimental data were analyzed using one-way ANOVA followed by Tukey’s multiple comparisons test or two-way ANOVA followed by Sidak’s multiple comparisons test. The Brown-Forsythe test was used to check for equal variances. Differences were considered significant when *p* < 0.05.

Please see Supplementary Methods for additional materials and methods.

## Results

### Low CYLD expression is associated with inferior overall survival in DLBCL and MCL

**Fig. 1:**
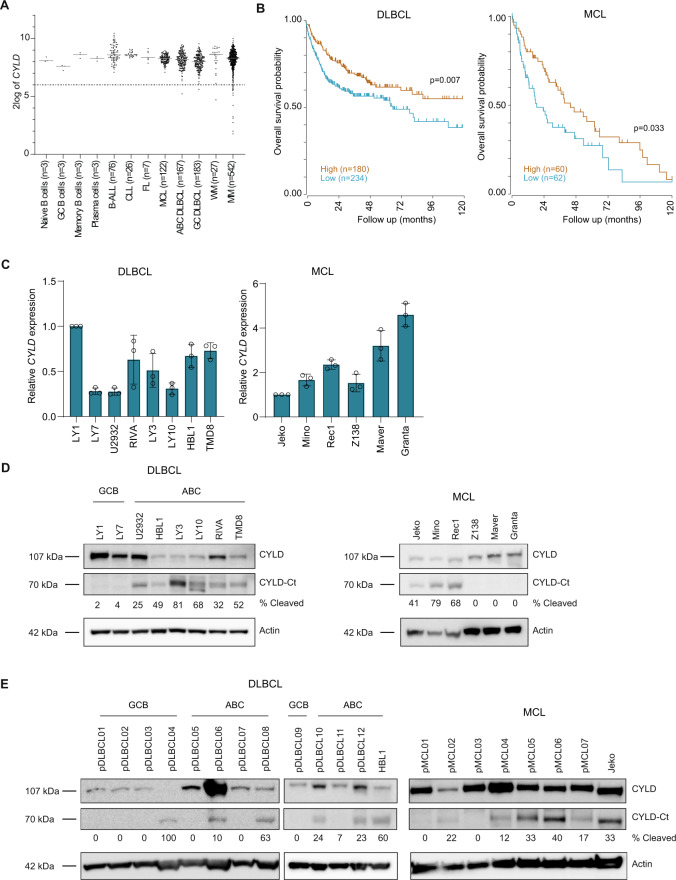
CYLD is variably expressed in B-cell non-Hodgkin lymphomas (B-NHLs). **A** *CYLD* mRNA expression analysis of publically available micro-array datasets of naïve B cells, GC (germinal center) B cells, memory B cells, plasma cells, B-cell acute lymphoblastic leukemia (B-ALL), chronic lymphocytic leukemia (CLL), follicular lymphoma (FL), mantle cell lymphoma (MCL), germinal center B-cell-like diffuse large B-cell lymphoma (GCB-DLBCL), activated B-cell-like diffuse large B-cell lymphoma (ABC-DLBCL), Waldenström’s macroglobulinemia (WM) and multiple myeloma (MM). The gray line represents the median expression value within each group. The dotted black line shows the threshold value (i.e., log-transformed probe intensity values of <2^6^). **B** Kaplan–Meier survival curve showing overall survival probability in *CYLD* high versus *CYLD* low expressing DLBCL and MCL patients. The cut off was based on the average *CYLD* expression within each cohort. The log-rank test was used to compare the survival distributions of the two groups. **C** RT-qPCR analysis of *CYLD* mRNA expression in DLBCL and MCL cell lines. *RPLP0* was used as an input control and data are normalized to *CYLD* expression in LY1 for DLBCL cell lines and Jeko for MCL cell lines. The mean ± SD of three independent experiments performed in triplicate is shown. **D** Immunoblot analysis of CYLD expression in DLBCL and MCL cell lines using an antibody raised against a C‐terminal epitope which detects full-length CYLD and a C‐terminal fragment of CYLD (CYLD‐Ct). β-actin was used as a loading control. **E** Immunoblot analysis of CYLD expression in primary DLBCL and MCL samples. β-actin was used as a loading control.

Analysis of gene expression microarray data shows that *CYLD* is expressed in normal B-cell subsets and all analyzed B-cell malignancies (Fig. 1A). In line with previous studies, the microarray data show that *CYLD* expression is lost in 3.3% (18/542) of MM. In contrast, *CYLD* expression was lost in only 0.6% (2/350) of DLBCL.

Interestingly, in line with its potential function as a tumor suppressor, low *CYLD* expression significantly correlates with poor overall survival in DLBCL and MCL. If DLBCL patients were separated into groups of low and high mRNA expression with average *CYLD* expression as cutoff, the median overall survival was below 6 years in the *CYLD* low patients, while it was over 10 years in the *CYLD* high patients (Fig. 1B). Of relevance, given the worse prognosis of ABC versus GCB DLBCL patients, no differential (i.e., lower) expression of *CYLD* was observed in ABC versus GCB DLBCL (Fig. 1A). When classified into ABC or GCB DLBCL, ABC DLBCL patients with low *CYLD* expression showed a trend toward worse overall survival (*p* = 0.085), while this was not the case for GCB DLBCL patients (Supplemental Fig. 1A). In MCL patients, the median overall survival was only 1.5 years in the *CYLD* low patients, compared to almost 4 years in the *CYLD* high patients (Fig. 1B). Taken together, our findings suggest that, although loss of *CYLD* is an uncommon mechanism in DLBCL and MCL, low *CYLD* expression is associated with inferior overall survival in DLBCL and MCL patients.

### Cleavage of CYLD is dependent on MALT1 protease activity

To further explore the potential role of CYLD as a tumor suppressor, we assessed *CYLD* mRNA and protein levels in a panel of DLBCL and MCL cell lines. In line with the primary lymphoma cases, we observed variable levels of *CYLD* mRNA expression in DLBCL and MCL cell lines (Fig. 1C). Interestingly, using an antibody raised against a C‐terminal epitope of CYLD, we detected a ~110 kDa protein corresponding with the anticipated molecular weight of CYLD in all cell DLBCL lines, but exclusively in the ABC DLBCL cell lines also a prominent protein of ~70 kDa was observed (Fig. 1D). This C-terminal CYLD fragment was also present in the MCL cell lines Jeko, Mino and Rec1, but was absent in Z138, Maver and Granta. In cell lines expressing only full-length CYLD, a strong correlation between *CYLD* mRNA expression and CYLD protein expression was observed (Supplemental Fig. 1B). Interestingly, in a panel of primary DLBCL and MCL cases we detected CYLD protein expression in all samples and a ~70 kDa cleaved CYLD fragment was observed in 1/5 GCB DLBCL (20%), 4/7 ABC DLBCL (57%) and 6/7 MCL cases (86%) (Fig. 1E).

**Fig. 2:**
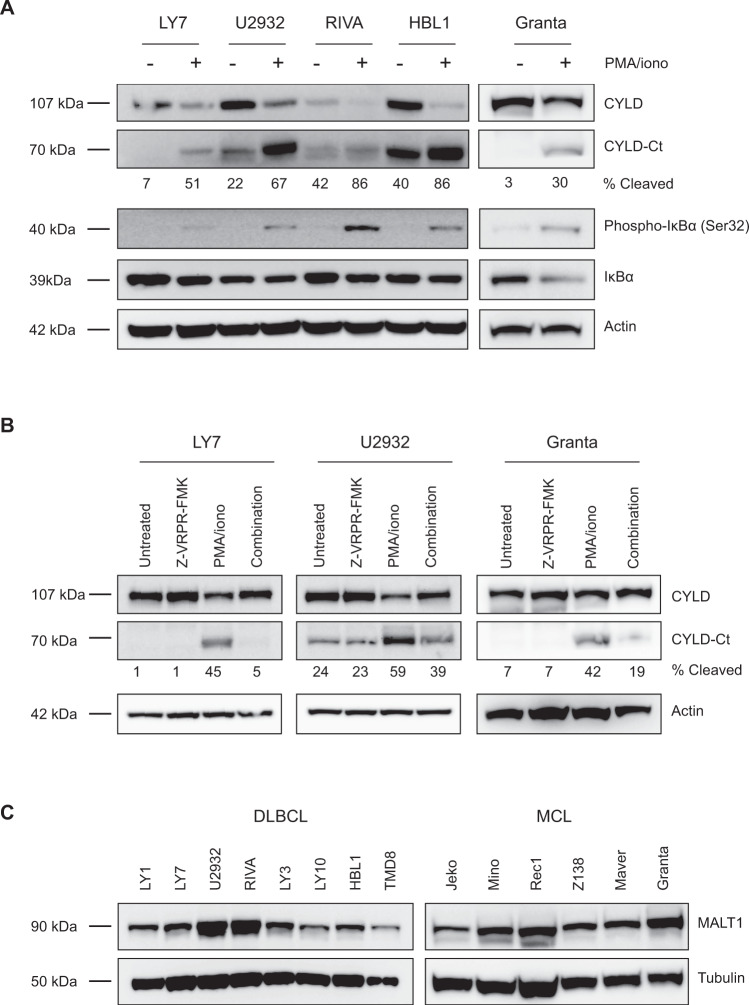
CYLD is cleaved by MALT1 protease. **A** Immunoblot analysis of CYLD cleavage following treatment with phorbol myristate acetate (PMA) and ionomycin. Cells were treated for 1 h with PMA (50 ng/ml) and ionomycin (1 µg/ml) as indicated. Phosphorylated IkBα (Ser32) and total IkBα were used as positive controls for PMA/ionomycin stimulation; β-actin was used as a loading control. **B** Immunoblot analysis of CYLD cleavage following treatment with PMA/ionomycin and/or MALT1 inhibitor Z-VRPR-FMK. Cells were pre-treated for 1 h with 75 µM Z-VRPR-fmk before incubation for 1 h with PMA (50 ng/ml) and ionomycin (1 µg/ml) as indicated. β-actin was used as a loading control. **C** Immunoblot analysis of MALT1 expression in DLBCL and MCL cell lines. β-tubulin was used as a loading control.

Previously, Staal et al. demonstrated that CYLD can be cleaved in T-cells by MALT1 at arginine 324 generating an N‐terminal fragment of 40 kDa and a C‐terminal fragment of 70 kDa [31]. To assess whether BCR signaling results in MALT1-mediated CYLD cleavage, we treated cell lines with phorbol myristate acetate (PMA) and ionomycin, which activate protein kinase C (PKC), a key intermediate of BCR-controlled MALT1 activation. In line with increased NF-κΒ activation, phosphorylation of IkB-α at serine 32 was increased in all cell lines and accordingly, total IkB-α levels were reduced as a consequence of proteasomal degradation. Moreover, in all cell lines treatment with PMA/ionomycin resulted in decreased levels of the 110 kDa full-length CYLD protein accompanied by increased levels of the 70 kDa C-terminal fragment (Fig. 2A). Importantly, pre-treatment with the MALT1 tetrapeptide protease inhibitor z‐VRPR‐fmk inhibits formation of the 70 kDa C-terminal fragment upon PMA/ionomycin treatment, demonstrating that CYLD cleavage is dependent on MALT1 proteolytic activity (Fig. 2B). The observation that CYLD is spontaneously cleaved in a subset of cell lines suggests that MALT1 is constitutively active in these cells (Fig. 1D). Since differences in MALT1 protein expression do not account for the observed differences in cleaved CYLD levels (Fig. 2C), this suggests that post-translational regulation of MALT1 activity, not MALT1 expression as such, determines CYLD cleavage in lymphoma cell lines.

### CYLD is constitutively cleaved in cell lines dependent on chronic BCR signaling

**Fig. 3:**
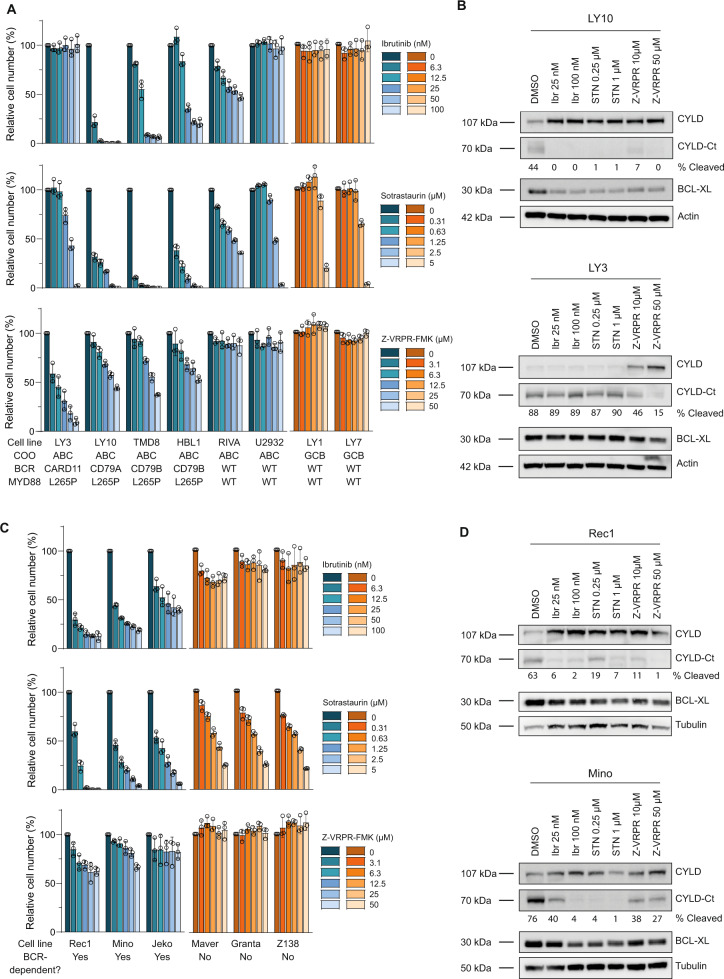
CYLD is constitutively cleaved in ABC DLBCL and BCR-dependent MCL cell lines. **A** Flow cytometric analysis of the number of viable cells, as determined by 7-AAD staining, after 5 days of treatment with indicated concentrations of BTK inhibitor Ibrutinib, PKC inhibitor Sotrastaurin or MALT1 inhibitor Z-VRPR-FMK. The number of viable cells was normalized to the vehicle-treated condition. Data are presented as mean ± SD of three independent experiments. **B** Immunoblot analysis of CYLD cleavage in DLBCL cell lines LY3 and LY10 using an antibody raised against a C‐terminal epitope which detects full-length CYLD and a C‐terminal fragment of CYLD (CYLD‐Ct). Cells were incubated with indicated concentrations of the different BCR signalosome inhibitors for 48 h. BCL-XL protein levels were determined as a positive control for efficacy of the inhibitors; β-actin was used as loading control. **C** Flow cytometric analysis of the number of viable cells, as determined by 7-AAD staining, after 7 days of treatment with indicated concentrations of BTK inhibitor Ibrutinib, PKC inhibitor Sotrastaurin or MALT1 inhibitor Z-VRPR-FMK. The number of viable cells was normalized to the vehicle-treated condition. Data are presented as mean ± SD of three independent experiments. **D** Immunoblot analysis of CYLD cleavage in MCL cell lines Rec1 and Mino using an antibody raised against a C‐terminal epitope which detects full-length CYLD and a C‐terminal fragment of CYLD (CYLD‐Ct). Cells were incubated with indicated concentrations of the different BCR signalosome inhibitors for 72 h. BCL-XL protein levels were determined as a positive control for efficacy of the inhibitors; β-tubulin was used as loading control.

ABC DLBCL tumors frequently harbor activating mutations in *CD79A/B* and *CARD11* which propagate ‘chronic’ BCR signaling [1, 3]. Cell lines harboring mutations in *CD79A* (LY10) or *CD79B* (HBL1 and TMD8) were highly sensitive to inhibition of BTK through treatment with ibrutinib and to inhibition of PKC through treatment with sotrastaurin, whereas LY3 cells harboring an activating mutation in *CARD11* downstream of BTK and PKC were resistant (Fig. 3A). In addition, blocking BCR signaling downstream of CARD11 by the MALT1 inhibitory peptide z‐VRPR‐fmk strongly reduced cell growth in all cell lines with *CARD11* or *CD79A/B* mutations (Fig. 3A).

Since MALT1 is a key signaling protein downstream of the BCR, we hypothesized that chronic BCR signaling contributes to CYLD cleavage in ABC DLBCL cell lines. Indeed, upon treatment with ibrutinib, sotrastaurin or z‐VRPR‐fmk, we observed a strong reduction in the 70 kDa cleavage product of CYLD in LY10, which was accompanied by accumulation of full-length CYLD (Fig. 3B). Similar results were obtained in ABC DLBCL cell lines U2932, HBL1 and TMD8 (Supplementary Fig. 2). Notably, in line with harboring an oncogenic *CARD11* mutation, LY3 only showed a reduction in CYLD cleavage upon incubation with z‐VRPR‐fmk but not with sotrastaurin or ibrutinib (Fig. 3B). In addition, except for ibrutinib and sotrastaurin treatment of LY3 (as expected), treatment with these BCR signalosome inhibitors substantially reduced expression of the NF-κB target and pro-survival protein BCL-XL in the ABC-DLBCL cell lines (Fig. 3B and Supplementary Fig. 2).

Recent studies showed that knockdown or pharmacological inhibition of central components of the BCR cascade was also toxic to a subset of MCL cell lines [4, 17]. In line with these studies, we confirmed that MCL cell lines Jeko, Mino and Rec1 strongly respond to treatment with ibrutinib, sotrastaurin or Z-VRPR-fmk (Fig. 3C). Moreover, inhibition of the BCR signaling cascade resulted in a substantial reduction of cleaved CYLD levels, accompanied by accumulation of full-length CYLD and downregulation of BCL-XL expression (Fig. 3D). In contrast, Z138, Maver and Granta were relatively resistant to inhibition of BTK, PKC or MALT1 suggesting that these cell lines do not depend on BCR signaling for their survival (Fig. 3C). Indeed, these cell lines did not show cleavage of CYLD, indicating a lack of MALT1 proteolytic activity (Fig. 1D). Hence, these data indicate that BCR signaling mediates survival as well as CYLD cleavage in BCR-dependent ABC DLBCL and MCL cell lines.

### CYLD represses cell growth and NF-κB activity

**Fig. 4:**
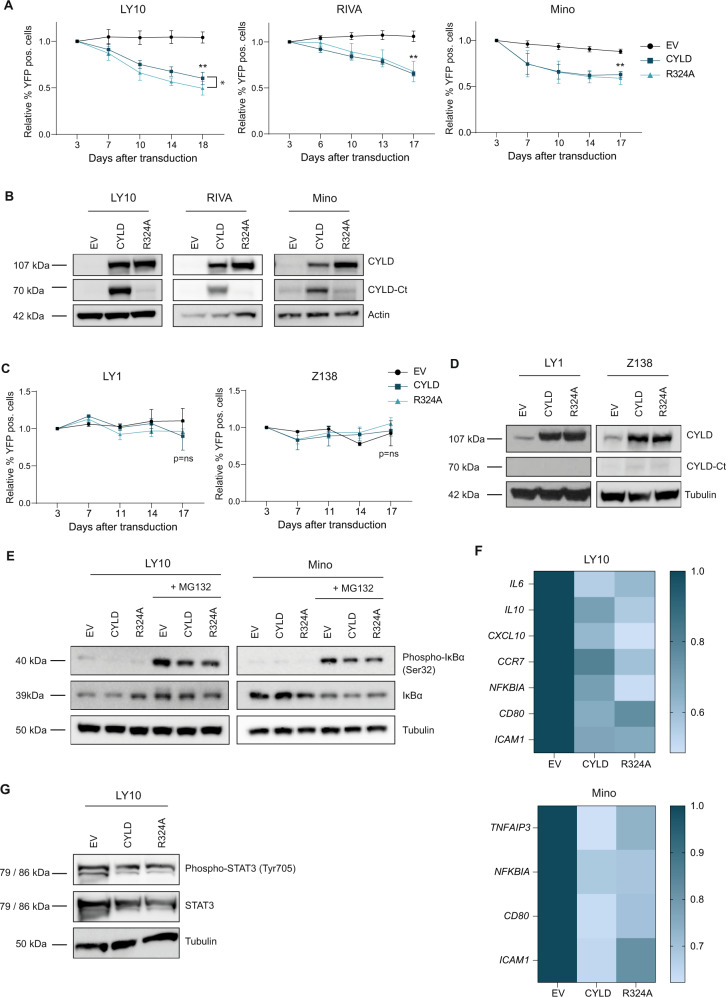
Ectopic expression of non-cleavable CYLD inhibits cell growth and NF-κB pathway activity. **A** Flow cytometric analysis of LY10, RIVA and Mino cells transduced with an empty vector (EV) or a CYLD (wildtype or R324A mutant) containing bicistronic vector co-expressed with YFP. The percentage of YFP positive cells was followed in time and plotted as the percentage of YFP^+^ cells, normalized to the value at day 3 following retroviral transduction. The mean ± SD of at least three independent transductions is shown. *P < 0.05; **P < 0.01 using 1-way ANOVA with Tukey’s multiple comparisons test. **B** Immunoblot analysis of CYLD in LY10, RIVA and Mino using an antibody raised against a C‐terminal epitope which detects full-length CYLD and a C‐terminal fragment of CYLD (CYLD‐Ct). Cells were transduced with an empty vector (EV) or an expression vector for CYLD (WT or non-cleavable R324A mutant) and sorted for YFP expression. β-actin was used as loading control. **C** Flow cytometric analysis of LY1 and Z138 cells transduced with an empty vector (EV) or a CYLD (wildtype or R324A mutant) containing bicistronic vector co-expressed with YFP. The percentage of YFP positive cells was followed in time and plotted as the percentage of YFP^+^ cells, normalized to the value at day 3 or day 4 following retroviral transduction. The mean ± SD of three independent transductions is shown. *P* > 0.05; ns (non-significant) using 1-way ANOVA with Tukey’s multiple comparisons test. **D** Immunoblot analysis of CYLD in LY1 and Z138 using an antibody raised against a C‐terminal epitope which detects full-length CYLD and a C‐terminal fragment of CYLD (CYLD‐Ct). Cells were transduced with an empty vector (EV) or an expression vector for CYLD (WT or non-cleavable R324A mutant) and sorted for YFP expression. β-tubulin was used as loading control. **E** Immunoblot analysis of (phosphorylated) IkBα in LY10 and Mino transduced with an empty vector (EV) or a CYLD (wildtype or R324A mutant) expressing vector. Three days after sorting cells were incubated with or without 5 µM proteasome inhibitor MG132 for 3 h before harvesting. β-tubulin was used as loading control. **F** Heatmap representing the RT-qPCR analysis of NF-κB target gene expression in LY10 and Mino transduced with an empty vector (EV) or an expression vector for CYLD (WT or non-cleavable R324A mutant). Cells were sorted for YFP expression and allowed to recover for 48 h before RNA isolation. *RPLP0* was used as an input control and data are normalized to the EV control expression levels. The mean of three independent experiments performed in triplicate is shown. **G** Immunoblot analysis of (phosphorylated) STAT3 in LY10 transduced with an empty vector (EV) or an expression vector for CYLD (WT or non-cleavable R324A mutant). β-tubulin was used as loading control.

To assess whether MALT1-mediated CYLD cleavage directly contributes to growth of BCR-dependent lymphoma cell lines, we generated a CYLD mutant that can no longer be cleaved by MALT1 (CYLD R324 A) [31]. Using a retroviral overexpression system, we expressed either wild type CYLD or CYLD R324A in a panel of lymphoma cell lines. CYLD expression clearly led to reduced survival in ABC DBCL cell lines LY10 and RIVA as well as the BCR-dependent MCL cell line Mino (Fig. 4A). A similar effect was observed for CYLD R324A, demonstrating that MALT1-mediated cleavage of CYLD is not required for the observed growth repression. Interestingly, in LY10 the inhibitory effect of CYLD expression was more pronounced upon overexpression of CYLD R324A, which is fully resistant to MALT1-mediated cleavage. Immunoblot analysis demonstrates that these cell lines lack sufficient MALT1 activity to effectively cleave all ectopically overexpressed wild type CYLD, explaining why ectopic expression of wild type CYLD also confers a prominent growth disadvantage (Fig. 4B). In the BCR-independent cell lines LY1 and Z138, CYLD expression did not affect cell survival (Fig. 4C). Moreover, immunoblot analysis shows that these cell lines indeed lack MALT1 protease activity as we only detected full-length CYLD upon expression of the wild type CYLD construct (Fig. 4D).

To study whether the CYLD-dependent growth inhibition is associated with reduced NF-κΒ activation, we performed immunoblot analysis to assess IkB-α phosphorylation. Since phosphorylation of IkB-α at serine 32 leads to its rapid proteosomal degradation, cells were co-incubated with proteasome inhibitor MG132. Indeed, we observed strong accumulation of phosphorylated IkB-α in LY10 and Mino incubated with MG132 (Fig. 4E). Intriguingly, phosphorylated IkB-α was strongly reduced in cells expressing CYLD (WT or R324A mutant), suggesting that CYLD represses NF-κΒ signaling upstream of the IKK complex. Analysis of *CYLD* mRNA ensured equal expression of wild type and mutated CYLD (Supplemental Fig. 3A). In addition, we performed RT-qPCR to determine expression of a panel of established NF-κΒ target genes, most of which are also implicated in lymphomagenesis [34]. In LY10, a ABC DLBCL cell line characterized by constitutive MALT1 activity, ectopic expression of CYLD reduced expression *of IL6, IL10, CXCL10, CCR7, NKFBIA, CD80* and *ICAM1* (Fig. 4F). In Mino, *IL6, IL10* and *CXCL10* were not expressed, but we did observe downregulation of *TNFAIP3, NFKBIA, CD80* and *ICAM1* upon expression of CYLD (WT or R324A mutant). Furthermore, in line with downregulation of *IL-6* and *IL-10*, we observed reduced phosphorylated STAT3 and total STAT3 levels in LY10 cells expressing CYLD (WT or R324A mutant). In accordance with a lack of IL-6 and IL-10 expression, we did not detect basal levels of phosphorylated STAT3 in Mino (Supplemental Fig. 3B).

CYLD has also been reported to act as a negative regulator of Wnt/β-catenin signaling in MM [35]; however, in DLBCL cell lines LY10 and RIVA, we hardly detected nuclear beta-catenin and in Mino nuclear beta-catenin levels were not affected by ectopic CYLD expression (Supplemental Fig. 3C). In addition, CYLD was previously shown to be involved in TCR-induced JNK phosphorylation [31]; however, we did not detect altered JNK phosphorylation upon ectopic expression of CYLD (Supplemental Fig. 3D). Altogether, our findings indicate that full-length CYLD represses growth of BCR-dependent cell lines, not by affecting Wnt/β-catenin or JNK signaling, but through suppression of NF-κΒ activity.

### MALT1-dependent cleavage suppresses activity of CYLD and promotes its proteosomal degradation

**Fig. 5:**
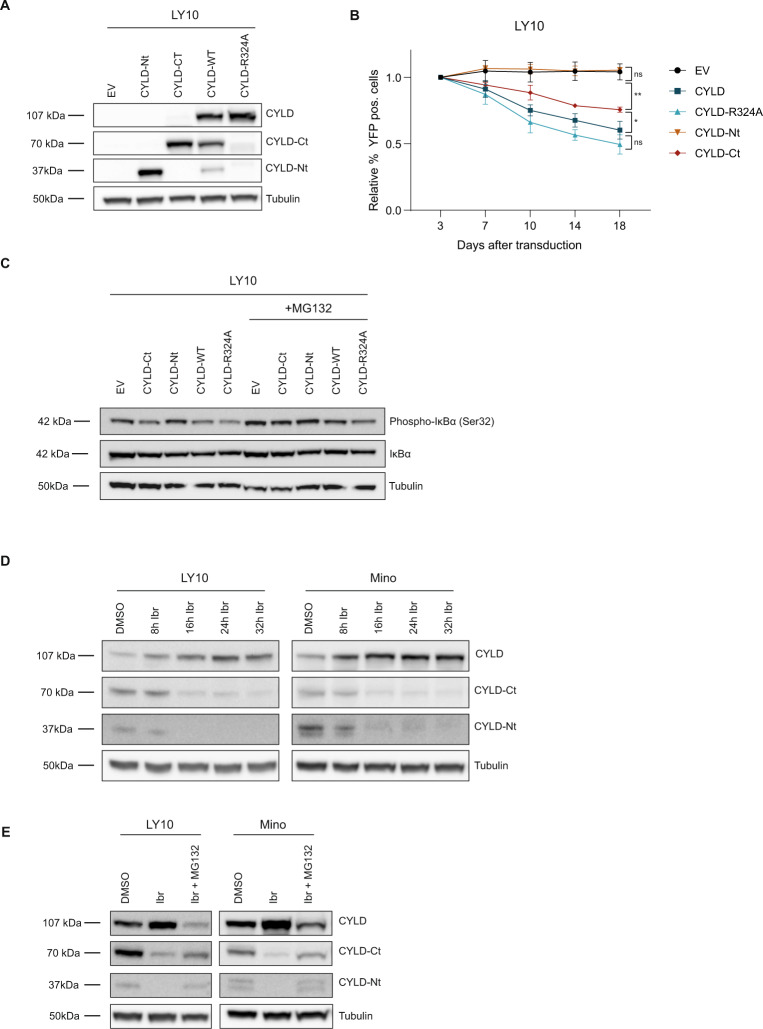
MALT1-dependent proteolytic cleavage inhibits activity and promotes proteasomal degradation of CYLD. **A** Immunoblot analysis of CYLD variants in LY10. Cells were transduced with an empty vector (EV) or an expression vector for CYLD (N-terminal fragment, C-terminal fragment, WT or non-cleavable R324A mutant) and sorted for YFP expression. CYLD was detected using an antibody raised against a C‐terminal epitope which detects full-length CYLD and a C‐terminal fragment of CYLD (CYLD‐Ct), or an antibody against an N‐terminal epitope for detection of the N-terminal fragment (CYLD-Nt). β-tubulin was used as loading control. **B** Flow cytometric analysis of LY10 cells transduced with an empty vector (EV) or a bicistronic expression vector for CYLD (N-terminal fragment, C-terminal fragment, WT or non-cleavable R324A mutant) co-expressing YFP. The percentage of YFP positive cells was followed in time and plotted as the percentage of YFP^+^ cells, normalized to the value at day 3 following retroviral transduction. The mean ± SD of four independent transductions is shown. *P* > 0.05; ns (non-significant); *P < 0.05; **P < 0.01 using 1-way ANOVA with Tukey’s multiple comparisons test. **C** Immunoblot analysis of (phosphorylated) IkBα in LY10 transduced with an empty vector (EV) or an expression vector for CYLD (N-terminal fragment, C-terminal fragment, WT or non-cleavable R324A mutant) expressing vector. Three days after sorting cells were incubated with or without 5 µM proteasome inhibitor MG132 for 3 h before harvesting. β-tubulin was used as loading control. **D** Immunoblot analysis of CYLD cleavage in LY10 and Mino. Cells were incubated with 100 nM ibrutinib for the indicated time points. β-tubulin was used as loading control. **E** Immunoblot analysis of endogenous CYLD cleavage in LY10 and Mino. Cells were incubated with 100 nM ibrutinib for 24 h in the presence or absence of 10 uM MG132. To prevent apoptosis, cell lines were co-incubated with 10 µM Q-VD-OPh (QVD). β-tubulin was used as loading control.

To assess the functionality of the CYLD fragments produced by MALT1-mediated cleavage, we ectopically expressed the resulting N-terminal or C-terminal fragment of CYLD, and for comparison also wild type CYLD or CYLD R324A (Fig. 5A and Supplemental Fig. 4A). Expression of the N-terminal CYLD fragment did not affect cell growth, while expression of the C-terminal CYLD fragment did reduce cell growth, albeit in LY10 to a lesser extent than expression of full-length CYLD. Likewise, expression of the N-terminal fragment in LY10 did not result in suppression of IkB-α phosphorylation, whereas IkB-α phosphorylation was slightly reduced in cells expressing the C-terminal fragment (Fig. 5C).

To further explore the functional implications of proteolytic cleavage of CYLD, we examined the fate of the endogenous C-terminal and N-terminal cleavage products. In both Mino and LY10, the C-terminal and N-terminal CYLD fragment were no longer detectable following 16 h of ibrutinib treatment, suggesting that these fragments are unstable (Fig. 5D). Addition of proteasome inhibitor MG132 to the ibrutinib-treated cells resulted in stabilization of both the C-terminal and N-terminal CYLD fragment (Fig. 5E). These data indicate that, following MALT1-mediated cleavage, the C-terminal and N-terminal CYLD fragment undergo subsequent degradation by the proteasome. In accordance, we observed accumulation of both the C-terminal and N-terminal CYLD fragment in LY10 and Mino solely incubated with the proteasome inhibitor MG132 (Supplemental Fig. 4C). Thus, after MALT1-mediated cleavage, the resulting CYLD fragments are quickly degraded by the proteasome.

### CYLD-deficient cells are less sensitive to BCR signalosome inhibitors

**Fig. 6:**
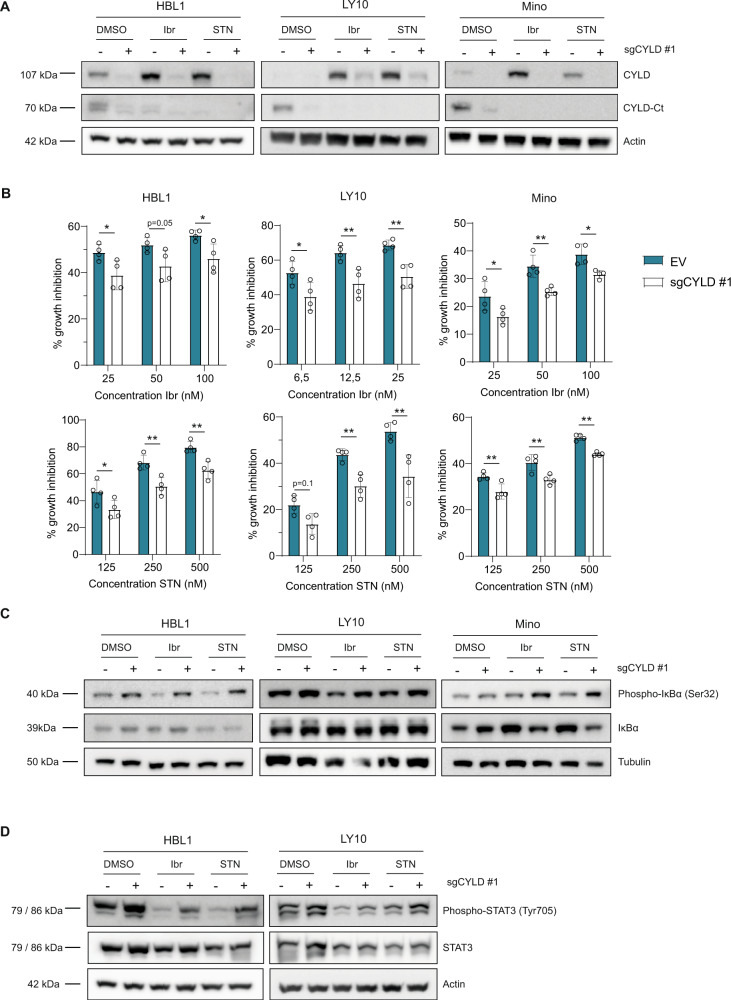
CYLD knockdown promotes cell growth and NF-κB activation. **A** Immunoblot analysis of CYLD in HBL1, LY10 and Mino transduced with lentiCRISPR-Cas9 (±sgCYLD) using an antibody raised against a C‐terminal epitope which detects full-length CYLD and a C‐terminal fragment of CYLD (CYLD‐Ct). Cells were treated with 50 nM BTK inhibitor Ibrutinib or 500 nM PKC inhibitor Sotrastaurin for 48 h as indicated. β-tubulin was used as loading control. **B** HBL1, LY10 and Mino transduced with lentiCRISPR-Cas9 without gRNA (empty vector; EV) or with sgCYLD were treated for 3 days with indicated concentrations of Ibrutinib or Sotrastaurin. The number of viable cells, as determined by 7-AAD staining, was normalized to the untreated condition. The mean ± SD of four independent experiments performed in triplicate is shown. *P < 0.05; **P < 0.01 using 2-way ANOVA with Sidak’s multiple comparisons test. **C** Immunoblot analysis of (phosphorylated) IkBα in HBL1, LY10 and Mino transduced with lentiCRISPR-Cas9 (±sgCYLD) treated for 48 h with 50 nM Ibrutinib or 500 nM Sotrastaurin as indicated. Cells were incubated with 5 µM proteasome inhibitor MG132 for 3 h before harvesting. β-tubulin was used as loading control. **D** Immunoblot analysis of (phosphorylated) STAT3 in LY10, HBL1 and Mino transduced with lentiCRISPR-Cas9 (±sgCYLD) treated 48 h with 50 nM Ibrutinib or 500 nM Sotrastaurin as indicated. β-actin was used as loading control.

Given the previously described function of CYLD as a negative regulator of NF-κB signaling [23–25], we investigated if loss of CYLD would be sufficient to augment NF-κB activity. For this purpose, we generated CYLD-deficient HBL1, LY10 and Mino cell lines using the CRISPR-Cas9 system. Considering that NF-κB is constitutively activated and that CYLD is partially cleaved in these cell lines, we treated the cells with BCR signalosome inhibitors to repress NF-κB activity and promote full-length CYLD accumulation. In line with our previous results (see Fig. 3B and D), treatment with ibrutinib and sotrastaurin strongly induced accumulation of full-length CYLD (Fig. 6A). This was largely impaired in CYLD-deficient cells, indicating efficient silencing of CYLD. Interestingly, whereas treatment with ibrutinib and sotrastaurin strongly repressed cell growth, as anticipated (see also Fig. 3A and C), this growth-inhibitory effect was significantly reduced in CYLD-deficient cells (Fig. 6B). Ensuring that the observed effects are on-target, similar effects were observed with a second CYLD gRNA (Supplementary Fig. 5A and B).

Notably, in HBL1, LY10 and Mino, ibrutinib and sotrastaurin induced a strong G1-arrest, which was partially restored in CYLD-deficient cells (Supplemental Fig. 6A and B), whereas cell viability was hardly affected, indicating that these inhibitors mainly arrest cell proliferation (Supplemental Fig. 6C and 6D).

To determine whether the enhanced cell proliferation in CYLD-deficient cells results from augmented NF-κB signaling, we next assessed IkB-α phosphorylation. Treatment with ibrutinib and sotrastaurin markedly reduced IkB-α phosphorylation, which was partially restored in CYLD-deficient cells (Fig. 6C and Supplemental Fig. 7). In line with the observed effects on NF-κB signaling, treatment with ibrutinib and sotrastaurin strongly reduced STAT3 phosphorylation, and this was partially overcome in CYLD-deficient cells (Fig. 6D). Since *STAT3* itself is a target gene of phosphorylated STAT3, these effects are also reflected by changes in total STAT3 protein. In accordance to Supplemental Fig. 3B, we did not observe basal levels of phosphorylated STAT3 in Mino (data not shown). Collectively, our findings demonstrate that loss of CYLD renders BCR-dependent lymphoma cell lines less sensitive to BCR pathway inhibition by ibrutinib and sotrastaurin, indicating that their inhibitory effects on proliferation and signaling are at least partially dependent upon CYLD.

## Discussion

**Fig. 7:**
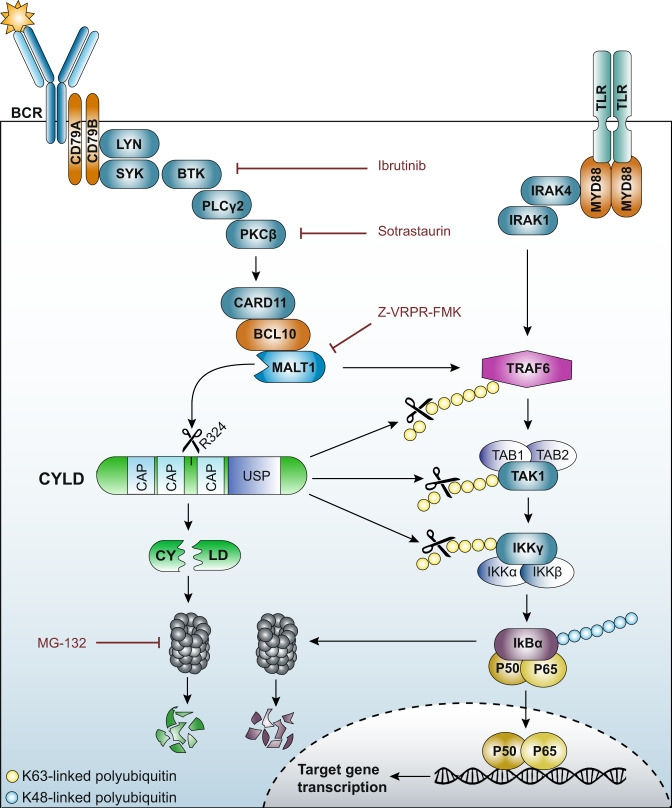
Model of the role of CYLD in NF-κB activation in B-cell lymphomas. Upon B-cell receptor (BCR) ligation, tyrosine residues within the ITAM motifs of CD79 are phosphorylated by the Src-family tyrosine kinase LYN leading to activation of spleen tyrosine kinase (SYK). Subsequently, Bruton’s tyrosine kinase (BTK) is activated and can then phosphorylate phospholipase Cγ2 (PLCγ2). PLCγ2 mediates the formation of second messengers that activate protein kinase Cβ (PKCβ). PKCβ phosphorylates caspase recruitment domain-containing protein 11 (CARD11) provoking a conformational change and allowing CARD11 to interact with B-cell lymphoma 10 (BCL10), and subsequently MALT1. MALT1 is a protease that cleaves various target proteins, including CYLD. In addition, oligomerized MALT1 functions as a scaffolding protein allowing recruitment of the E3 ubiquitin ligase tumor necrosis factor receptor-associated factor 6 (TRAF6). In parallel, Toll-like receptor (TLR) engagement results in MyD88-dependent recruitment of IL-1 receptor-associated kinase-4 (IRAK4) and subsequently IRAK1. IRAK4 phosphorylates IRAK1, which then can associate with TRAF6. BCR/TLR-activated TRAF6 promotes Lys-63-linked ubiquitination of TRAF6 itself as well as transforming growth factor beta-activated kinase 1 (TAK1) and NEMO/IKK-γ. Ubiquitinated TRAF6 binds to adaptor proteins TAB1/2/3, leading to the recruitment and auto-phosphorylation of TAK1. Ubiquitination of NEMO/IKK-γ mediates the recruitment of the IKK subunits to the TAK1/TAB complex, thereby facilitating the phosphorylation of IKK-β by TAK1. IKK-β then phosphorylates IκBα resulting in Lys-48-polyubiquitination and subsequent proteasomal degradation which allows NF-κB dimers to translocate to the nucleus. The deubiquinating enzyme CYLD consists of three conserved cytoskeleton-associated protein glycine-rich (CAP-Gly) domains and a C-terminal catalytic ubiquitin-specific protease (USP) domain that is able to hydrolyze lysine 63-linked ubiquitin chains. CYLD can hydrolyze Lys-63-linked polyubiquitin chains of TRAF6, TAK1 and/or NEMO/IKK-γ, thereby suppressing NF-κB activation. Accordingly, MALT1-dependent cleavage of CYLD substantially reduces its functionality and initiates its proteasomal degradation, thereby promoting cell growth and NF-κB activation.

CYLD has been implicated in the pathogenesis of many malignancies, including breast, colon, liver and skin cancers [28, 36–38]. Our study reveals a tumor suppressive function of CYLD in the pathogenesis of DLBCL and MCL. First, our microarray analysis showed that high *CYLD* expression correlates with improved overall survival in both DLBCL and MCL patients. Previous studies have established that high *CYLD* expression is also associated with improved overall survival in CLL and MM [35, 39]. Interestingly, in MM, a malignancy of plasma cells, CYLD expression is frequently lost through deletions or inactivating mutations [26, 40]. These genomic aberrations hardly occur in DLBCL and MCL, suggesting an important role for other post-translational or transcriptional control mechanisms [27]. At the post-translational level, phosphorylation of CYLD at serine 418 by IKK has been demonstrated to reduce its deubiquitinase activity [28, 29, 41]. In addition, CYLD can be cleaved by caspase 8 at aspartate 215 promoting its degradation, as well as by (para)caspase MALT1 at arginine 324 resulting in its proteolytic inactivation [30, 31]. In various in vitro models, CYLD has been demonstrated to negatively regulate NF-κB activation and interact with many proteins that are essential in the signal transduction cascade mediating NF-κB activation [23–25, 35, 37]. This is in accordance with our findings showing that silencing of CYLD promotes NF-κB activation and cell growth and, conversely, ectopic expression of CYLD represses NF-κB signaling and cell growth in BCR-dependent lymphoma cell lines. In addition, our data demonstrate that MALT1-dependent cleavage of CYLD substantially reduces its functionality and, moreover, initiates its proteosomal degradation. Taken together, our data revealed that (1) CYLD is cleaved by MALT1, (2) these MALT1-mediated cleavage products of CYLD undergo rapid proteasomal degradation (inactivation), and (3) CYLD represses NF-κB activity and cell growth; hence, MALT-mediated cleavage of CYLD promotes NF-κB activity and growth of aggressive B-cell receptor-dependent lymphomas. Notably, this may also affect NF-κB activity controlled by, e.g., TLR/MYD88-signaling (summarized in Fig. 7).

CYLD consists of three conserved cytoskeleton-associated protein glycine-rich (CAP-Gly) domains and a C-terminal catalytic ubiquitin-specific protease (USP) domain that is able to hydrolyze lysine 63-linked ubiquitin chains (Fig. 7). The first and second CAP-Gly domains bind to microtubules, which might be required for optimal localization and interaction of CYLD with its interaction partners such as TRAF2, TRAF6 or TAK1 [23–25, 42], whereas the third CAP-Gly domain interacts with NEMO/IKKγ [43, 44]. Furthermore, Elliot et al. recently demonstrated that both the second and third CAP-Gly domain contain ubiquitin-binding domains and can therefore contribute to CYLD deubiquitinase activity [45]. As anticipated, our data show that the N-terminal fragment (lacking the third CAP-Gly and USP domain) is unable to repress cell growth. The observed ability of the C-terminal fragment to partially repress growth of LY10 cells suggests that, whereas the first and/or second CAP-Gly domain are required for optimal deubiquitinase activity, ectopic (high) overexpression of the C-terminal fragment may to some extent overcome the dependence upon the localization domains. Importantly, however, in addition to reduced functionality, our current findings demonstrate that the MALT1-produced endogenous CYLD fragments are rapidly degraded by the proteasome. Previous studies demonstrated that the E3 ubiquitin ligases TRIM47 and MIB2 are involved in proteosomal degradation of full-length CYLD [46, 47]. Interestingly, TRIM47 predominantly interacts with the N-terminal CAP-Gly domains, whereas MIB2 preferentially interacts with the third CAP-Gly domain, suggesting that these ligases could be involved in degradation of the CYLD fragments generated upon MALT1-mediated cleavage.

Next to CYLD, several other MALT1 substrates have been identified, but the complete role of the MALT1 protease activity in lymphomagenesis remains incompletely understood. MALT1-dependent cleavage of A20 and RelB, as well as MALT1 auto-proteolysis, have been implicated in fine-tuning NF-κB activation [13, 15, 48]. Since CYLD and A20 are both MALT1 targets which negatively regulate NF-κB signaling by deconjugating ubiquitin chains of largely overlapping substrates, it is remarkable that genetic aberrations in *CYLD* are rare, while deletions/mutations of *TNFAIP3*/A20 occur in over 30% of various B-cell malignancies [27, 49, 50]. This suggests that CYLD may have non-redundant, essential functions other than NF-κB suppression. Interestingly, Stegmeier et al., demonstrated that CYLD deubiquitinase activity is required for efficient mitotic entry, independent of its role in canonical NF-κB signaling [51]. Later studies showed that CYLD directly interacts with microtubules promoting their assembly and stability, which is essential for cell division [43, 52]. In line with an important role in cell cycle progression, CYLD is constitutively expressed in most cell types, albeit at a low level [53]. In contrast, A20 expression is mostly low/absent under basal conditions, but can be strongly upregulated in response to various stimuli [53–55]. The complex role of CYLD in cell cycle regulation and how this relates to its tumor suppressive functions remains to be fully elucidated.

Our current findings underline that MALT1 inhibitors are promising therapeutic agents for B-cell lymphomas that are dependent on chronic BCR signaling. The MALT1 inhibitor z‐VRPR‐fmk effectively inhibits ABC DLBCL growth in vitro and in vivo, but is presumably unsuitable for clinical applications as a consequence of its large size and relatively poor cell permeability [16, 18]. The first small molecule inhibitor irreversibly targeting MALT1, MI-2, showed both safety and efficacy in mouse models [18]. In addition, phenothiazine derivatives, which reversibly inhibit MALT1, were shown to strongly represses ABC DLBCL growth in vitro and in vivo [21, 56]. The first clinical trials using the MALT1 inhibitor JNJ-67856633 are currently ongoing in patients with non-Hodgkin’s Lymphoma and CLL (Clinical trials.gov; NCT03900598 and NCT04876092). In addition, ONO-7018 (formerly known as CTX-177) was demonstrated to be effective in ABC DLBCL and MCL models in vitro and in vivo and will soon enter clinical trials [57, 58] (Clinical trials.gov; NCT05515406).

In addition, our findings suggest that inhibition of MALT1-mediated CYLD cleavage, through other BCR signalosome inhibitors such as ibrutinib and sotrastaurin, contributes to the anti-tumor effects of these drugs. These findings are of great interest in the context of (pre-)clinical studies showing that ibrutinib and sotrastaurin are highly effective in ABC DLBCL cases harboring mutations that promote chronic BCR signaling [9, 59–62].

Altogether, our findings establish an important role for MALT1-mediated CYLD cleavage in BCR signaling, canonical NF-κB activity, and consequently cell growth of BCR-dependent lymphomas, thereby providing novel insights into targeting MALT1 protease activity and ubiquitination enzymes as a promising therapeutic approach for these aggressive lymphomas.

## Supplementary information

Supplemental Methods and Supplemental Figures

## Data Availability

The datasets generated during and/or analyzed during the current study are available from the corresponding author on reasonable request.
