# Supplementary material for: MALT1-dependent cleavage of CYLD promotes NF-κB signaling and growth of aggressive B-cell receptor-dependent lymphomas
Source: Blood Cancer J. 2023 Mar 15;13(1):37. doi: 10.1038/s41408-023-00809-7 (PMC10017792; doi:10.1038/s41408-023-00809-7)
Supplement: Supplementary file 1 — Supplemental Methods and Supplemental Figures [file 41408_2023_809_MOESM1_ESM.pdf]

Supplemental Methods and Supplemental Figures

Supplemental Methods

Microarray analyses

All microarray datasets were generated using the Affymetrix Human Genome U133 Plus 2.0 Array and are publically available through the NCBI Gene Expression Omnibus. Datasets analyzed were: GSE12366 (B-cell subsets), GSE11877 (B-ALL), GSE21029 (CLL), GSE16024 (FL), GSE93291 (MCL), GSE10846 (DLBCL), GSE9656 (WM) and GSE2658 (MM). Data analysis and visualization was performed using R2: Genomics Analysis and Visualization Platform (http://r2.amc.nl).

Cell lines and reagents

The MCL cell lines JeKo-1, Granta-519, Maver-1, Z-138, Rec-1 and Mino and the DLBCL cell lines OCI-LY1, OCI-LY3 and OCI-LY7 were cultured in IMDM supplemented with 10% FCS. OCI-LY10 was cultured in IMDM supplemented with 20% human serum (Sigma Aldrich, Saint Louis, Missouri, USA). U2932 and RIVA were cultured in RPMI-1640 supplemented with 10% FCS. HBL1 and TMD8 were cultured in RPMI-1640 supplemented with 20% FCS. Cell Line authentication was routinely performed using Short Tandem Repeat DNA profiling (PowerPlex 16, Promega, Leiden, The Netherlands) and cell lines were frequently tested for mycoplasma contamination using RT-qPCR.

The small molecule inhibitors Ibrutinib and Sotrastaurin and proteasome inhibitor MG132 were purchased from Selleckchem (Houston, Texas, USA); the MALT1 inhibitor Z-VRPR-FMK was purchased from Enzo Life Sciences (Farmingdale, New York, USA). Phorbol myristate acetate (PMA) and ionomycin were both purchased from Sigma Aldrich. Q-VD-OPh was purchased from MedChemExpress (Princeton, New Jersey, USA).

Transfection and transduction

To generate CYLD knockout cell lines, we inserted a single guide RNA targeting CYLD (sgRNA1: ATGGGAAGGACGATTCTGCC, sgRNA2: TGAGACTGAATGGTAAAGAG) into pL-CRISPR.EFS.GFP (Addgene plasmid #57818). For retroviral overexpression of CYLD we subcloned CYLD (NM_015247) from Flag-HA-CYLD (Addgene plasmid #22544) into LZRS-IRES-YFP, which was kindly provided by Dr. H. Spits. We employed the QuikChange II Site-Directed Mutagenesis Kit (Agilent, Santa Clara, California, USA) to generate a CYLD R324A mutant construct according to the manufacturer's instructions. To generate CYLD-Ct and CYLD-Nt expression constructs, we employed a PCR-based cloning strategy using LZRS-(HA)-CYLD-YFP as target sequence. We designed primers in which a start/stop codon and compatible restriction sites (BamHI and NotI) were embedded. The following primer pairs were used:

CYLD-Nt forward (5’-AGTAGGATCCACCATGGCTTACCCATAC-3’);
CYLD-Nt reverse (5’-TACTGCGGCCGCTTATCTTGACATAAAGGCAAG-3’);

CYLD-Ct forward (5’-AGTAGGATCCACCATGGGTGTTGGGGACAAAGGT-3’);
CYLD-Ct reverse (5’-TACTGCGGCCGCTTATTTGTACAAACTCATTGT-3’).

Following digestion, the PCR products were subcloned into LZRS-IRES-YFP. Sanger sequencing was used to validate the generated expression constructs.

Immunoblotting

Whole cell lysates were generated by lysing cells in RIPA buffer (20 mM Tris-HCl pH 7.4, 150 mM NaCl, 1% NP-40, 0.5% Sodium deoxycholate, 0.1% SDS, 5 mM EDTA, 10% glycerol) supplemented with protease inhibitors (Roche, Basel, Switzerland) and phosphatase inhibitors (Sigma Aldrich). Nuclear fractionation was performed using the Nuclear/Cytosol Fractionation Kit (BioVision, Milpitas, California, USA) according to the manufacturer's instructions. The primary antibodies used were: mouse anti-β-tubulin (clone D66, Sigma Aldrich), mouse anti-β-actin (clone AC-15, Sigma-Aldrich), mouse anti-CYLD (C-terminus) (clone E-10, Santa Cruz, Dallas, Texas, USA), mouse anti-MALT1 (clone B-12, Santa Cruz), rabbit anti-BCL XL (clone

54H6, Cell Signaling Technology, Danvers, Massachusetts, USA), rabbit anti-phospho-IκBα (Ser32) (clone 14D4, Cell Signaling Technology), rabbit anti-IκBα (clone 44D4, Cell Signaling Technology), rabbit anti-phosho-STAT3 (Tyr705) (Cell Signaling Technology), mouse-anti-STAT3 (clone 124H6, Cell Signaling Technology), rabbit anti-CYLD (N-terminus) (clone D1A10, Cell Signaling Technology), mouse anti-beta-catenin (clone 14, BD Biosciences, Franklin Lakes, New Jersey, USA), rabbit anti-TBP (Abcam, Cambridge, United Kingdom) and rabbit anti-phospho-SAPK/JNK (Thr183/Tyr185) (Cell Signaling Technology). Secondary antibodies used were anti-mouse-HRP or anti-rabbit-HRP (both DAKO, Agilent). Quantification of immunoblot membranes was performed using Image Lab software (Bio-Rad Laboraties, Hercules, California, USA).

RNA isolation, cDNA synthesis and RT-qPCR

Total RNA was isolated using TRI-reagent according to the manufacturer's protocol (Sigma Aldrich). RNA was converted to cDNA using oligo(dT) primers for 1 hour at 37 °C. RT-qPCRs were performed using Sensifast (Bioline, London, United Kingdom) on a on a Lightcycler 480 (Roche). Expression levels were normalized relative to expression of control gene *RPLP0*. The following primer pairs were used:

CYLD forward (5′– TGCAGGCTGTACGGATGGAACCT –3′);
CYLD reverse (5′–TCCTGATGCAGCCTCCACCT –3′);

IL6 forward (5′– GACTTGCCTGGTGAAAATCATCACTG –3′);
IL6 reverse (5′– GGGTCAGGGGTGGTTATTGCATC –3′);

IL10 forward (5′– TTACCTGGAGGAGGTGATGC –3′);
IL10 reverse (5′– GGCCTTGCTCTTGTTTTCAC–3′);

CXCL10 forward (5′– GTGGCATTCAAGGAGTACCTC –3′);
CXCL10 reverse (5′– TGATGGCCTTCGATTCTGGATT–3′);

TNF forward (5′– CTCTTCTGCCTGCTGCACTTTG –3′);
TNF reverse (5′– ATGGGCTACAGGCTTGTCACTC–3′);

CCR7 forward (5′– CAACATCACCAGTAGCACCTGTG –3′);
CCR7 reverse (5′– TGCGGAACTTGACGCCGATGAA–3′);

ICAM1 forward (5′– CATCTACAGCTTTCCGGCGCCC –3′);
ICAM1 reverse (5′– AGAAGCTGCGCCCGTTGTCC–3′);

CD80 forward (5′– TAGATGCGAGTTTGTGCCAG –3′);
CD80 reverse (5′– GCTGGCTGGTCTTTCTCACT–3′);

RPLP0 forward (5′– GCTTCCTGGAGGGTGTCCGC –3′);
RPLP0 reverse (5′– TCCGTCTCCACAGACAAGGCCA–3′).

Supplemental Figures

Supplemental Figure 1

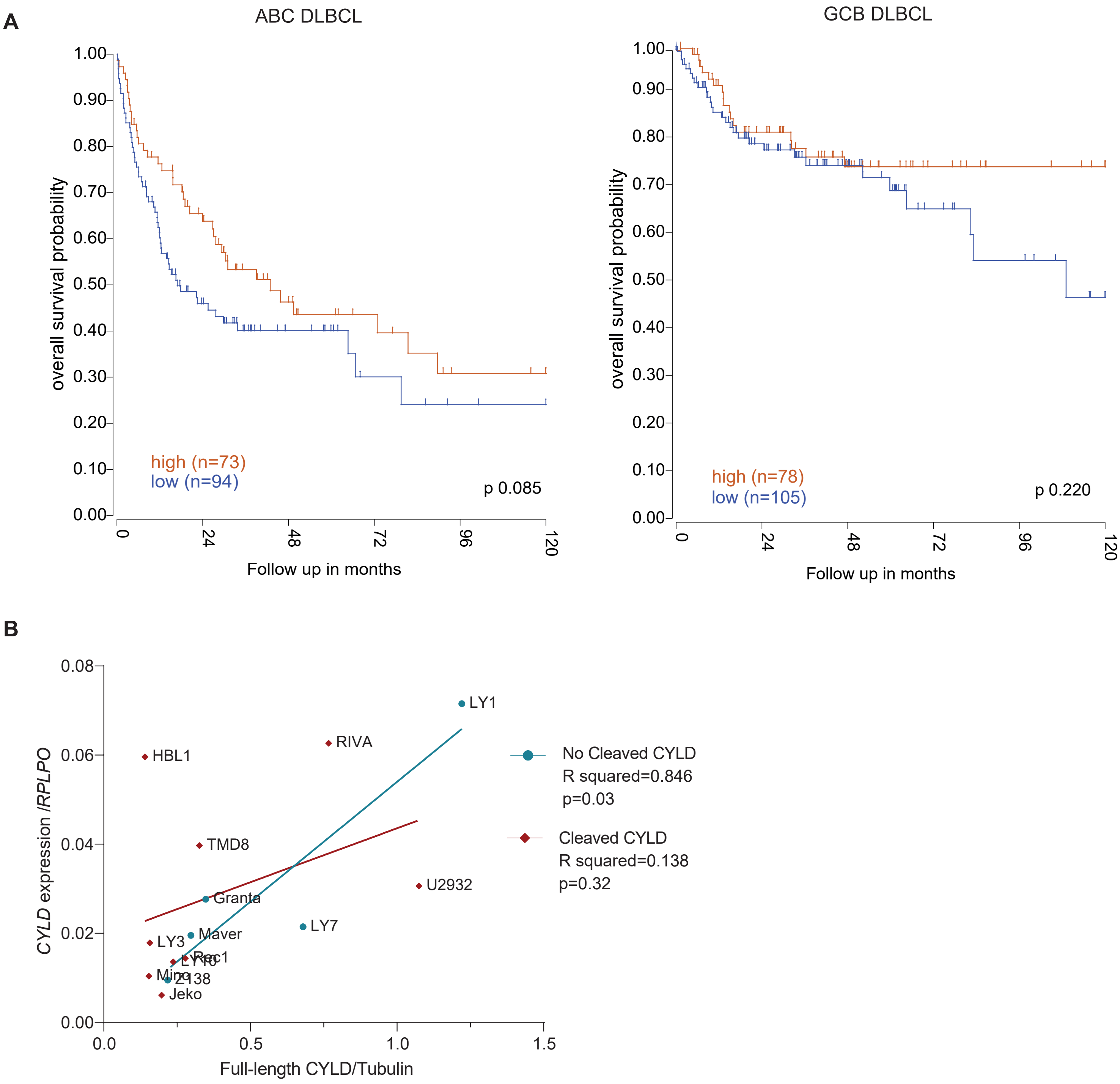

Supplementary Figure 1. (A) Kaplan-Meier survival curve showing overall survival probability in *CYLD* high versus *CYLD* low expressing ABC and GCB DLBCL patients. The cut off was based on the average *CYLD* expression within each cohort. The log-rank test was used to compare the survival distributions of the two groups. (B) Scatterplot with simple linear regression model assessing the correlation between *CYLD* mRNA as determined in Figure 1C and full-length CYLD protein expression as determined in Figure 1D. Cell lines expressing only full-length CYLD are visualized in blue. Cell lines expressing a cleaved CYLD fragment are visualized in red.

Supplemental Figure 2

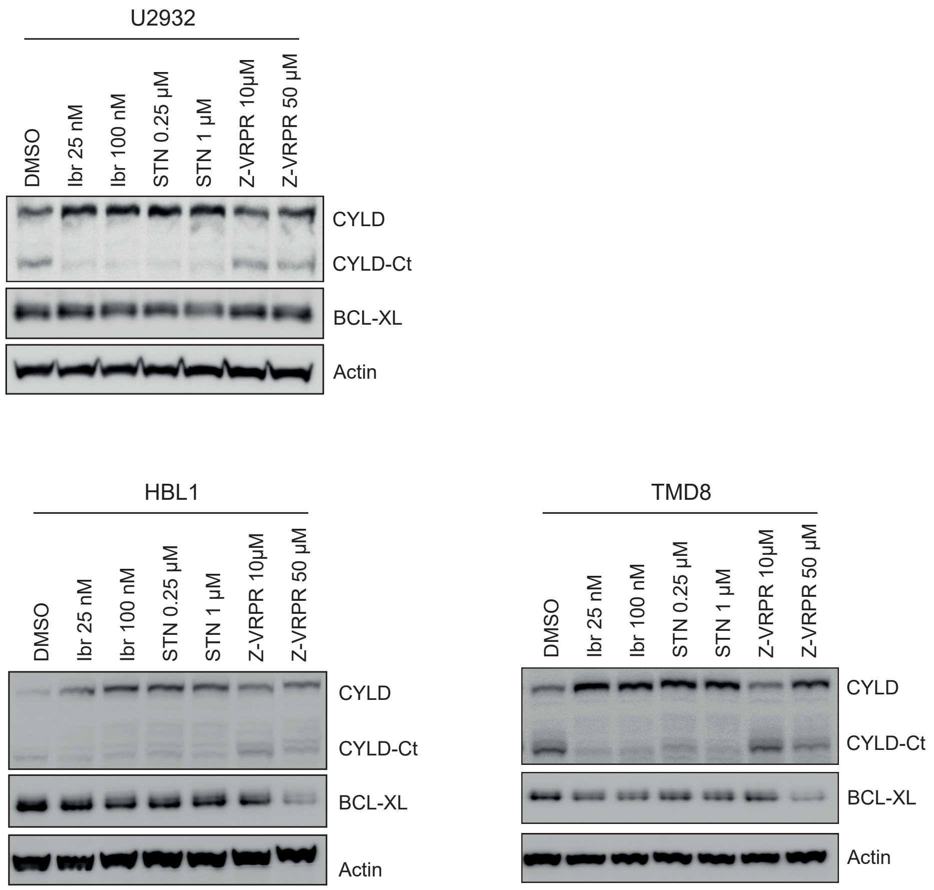

Supplementary Figure 2. Immunoblot analysis of CYLD cleavage in DLBCL cell lines U2932, HBL1 and TMD8 using an antibody raised against a C-terminal epitope which detects full-length CYLD and a C-terminal fragment of CYLD (CYLD-Ct). Cells were incubated with indicated concentrations of the different BCR signalosome inhibitors for 48 hours. BCL-XL protein levels were determined as a positive control for efficacy of the inhibitors; β-actin was used as loading control.

Supplemental Figure 3

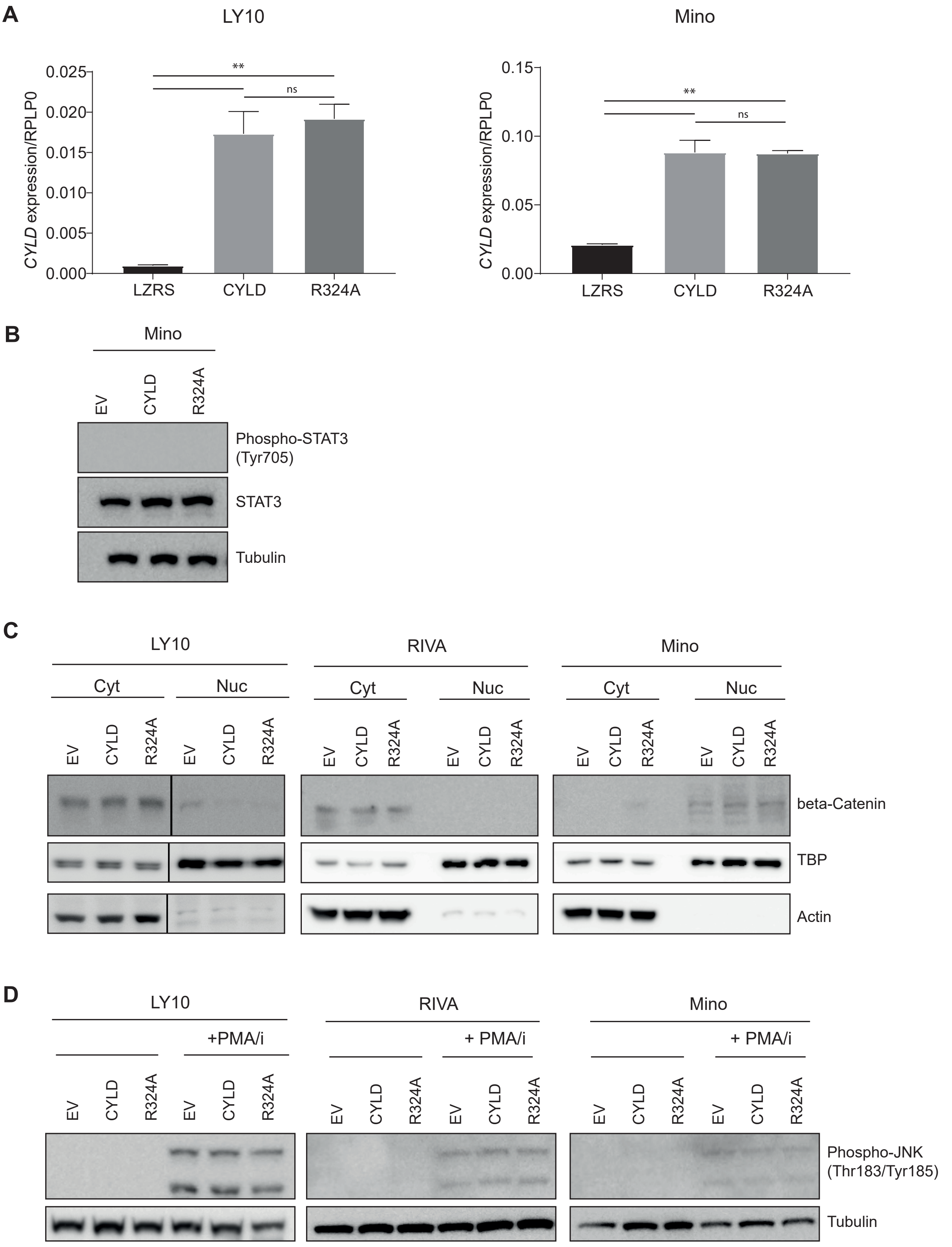

Supplementary Figure 3. (A) RT-qPCR analysis of CYLD expression in LY10 and Mino transduced with an empty vector (LZRS) or an expression vector for CYLD (WT or non-cleavable R324A mutant). Cells were sorted for YFP expression and allowed to recover for 48 hours before RNA isolation. RPLP0 was used as an input control. The mean ± SD of one representative experiment is shown. **P<0.01; P>0.05; ns (non-significant) using 1-way ANOVA with Tukey's multiple comparisons test. (B) Immunoblot analysis of (phosphorylated) STAT3 in Mino transduced with an empty vector (EV) or an expression vector for CYLD (WT or non-cleavable R324A mutant) β-tubulin was used as loading control. (C) Immunoblot analysis of beta-catenin levels following nuclear fractionation in LY10, RIVA and Mino transduced with an empty vector (EV) or a CYLD (wildtype or R324A mutant) expressing vector. TBP was used as a loading control for the nuclear fraction; β-actin was used as loading control for the cytosolic fraction. (D) Immunoblot analysis of phosphorylated JNK (Thr183/Tyr185) levels following stimulation of 1 hour with PMA (50 ng/ml) and ionomycin (1 μg/ml) in LY10, RIVA and Mino transduced with an empty vector (EV) or a CYLD (wildtype or R324A mutant) expressing vector. β-tubulin was used as loading control.

Supplemental Figure 4

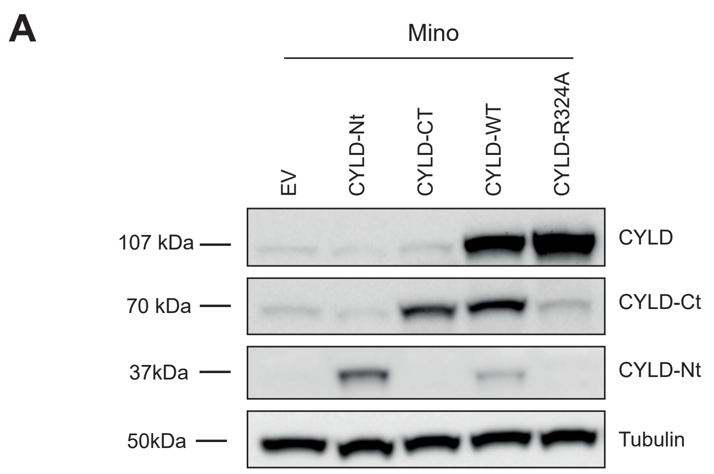

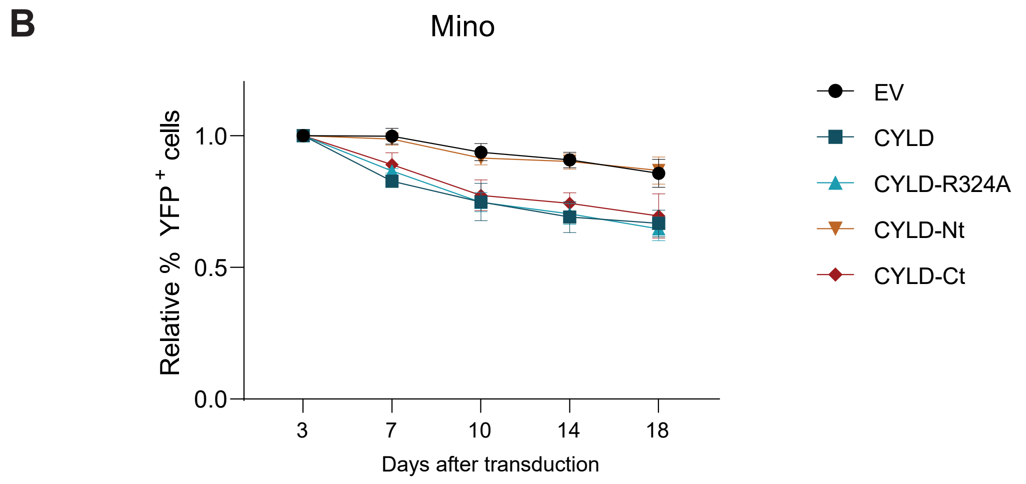

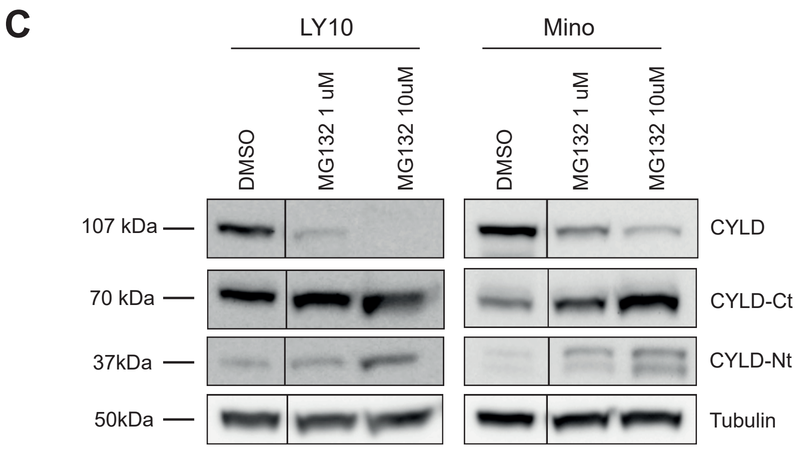

Supplementary Figure 4. (A) Immunoblot analysis of CYLD variants in Mino. Cells were transduced with an empty vector (EV) or an expression vector for CYLD (N-terminal fragment, C-terminal fragment, WT or non-cleavable R324A mutant) and sorted for YFP expression. CYLD was detected using an antibody raised against a C-terminal epitope which detects full-length CYLD and a C-terminal fragment of CYLD (CYLD-Ct), or an antibody against an N-terminal epitope for detection of the N-terminal fragment (CYLD-Nt). β-tubulin was used as loading control. (B) Flow cytometric analysis of Mino transduced with an empty vector (EV) or a bicistronic expression vector for CYLD (N-terminal fragment, C-terminal fragment, WT or non-cleavable R324A mutant) co-expressing YFP. The percentage of YFP positive cells was followed in time and plotted as the percentage of YFP+ cells, normalized to the value at day 3 following retroviral transduction. The mean ± S.E.M. of three independent transductions is shown. (C) Immunoblot analysis of CYLD cleavage in LY10 and Mino. Cells were incubated with indicated concentrations of MG132 for 24 hours. To prevent apoptosis, cell lines were co-incubated with 10μM Q-VD-OPh (QVD). CYLD was detected using an antibody raised against a C-terminal epitope which detects full-length CYLD and a C-terminal fragment of CYLD (CYLD-Ct). A second antibody raised against an N-terminal epitope was used for detection of the N-terminal fragment (CYLD-Nt). β-tubulin was used as loading control.

Supplemental Figure 5

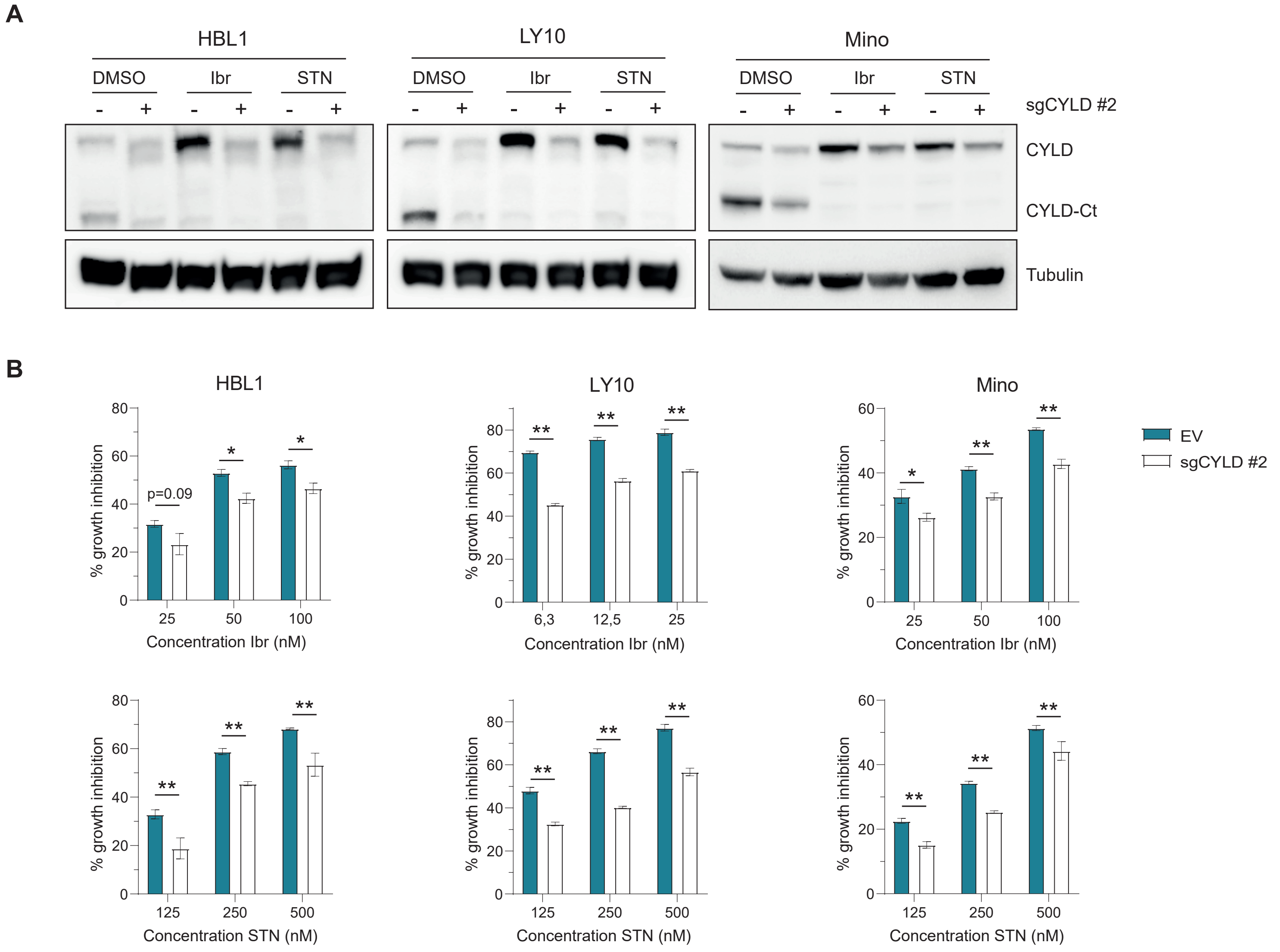

Supplementary Figure 5. (A) Immunoblot analysis of CYLD in HBL1, LY10 and Mino transduced with lentiCRISPR-Cas9 (+/- sgCYLD) using an antibody raised against a C-terminal epitope which detects full-length CYLD and a C-terminal fragment of CYLD (CYLD-Ct). Cells were treated with 50 nM BTK inhibitor Ibrutinib or 500 nM PKC inhibitor Sotrastaurin for 48 hours as indicated. β-tubulin was used as loading control. (B) HBL1, LY10 and Mino transduced with lentiCRISPR-Cas9 without gRNA (empty vector; EV) or with sgCYLD were treated for 3 days with indicated concentrations of Ibrutinib or Sotrastaurin. The number of viable cells, as determined by 7-AAD staining, was normalized to the untreated condition. The mean ± SD of one representative experiment is shown. *P<0.05; **P<0.01 using 2-way ANOVA with Bonferroni's multiple comparisons test.

Supplemental Figure 6

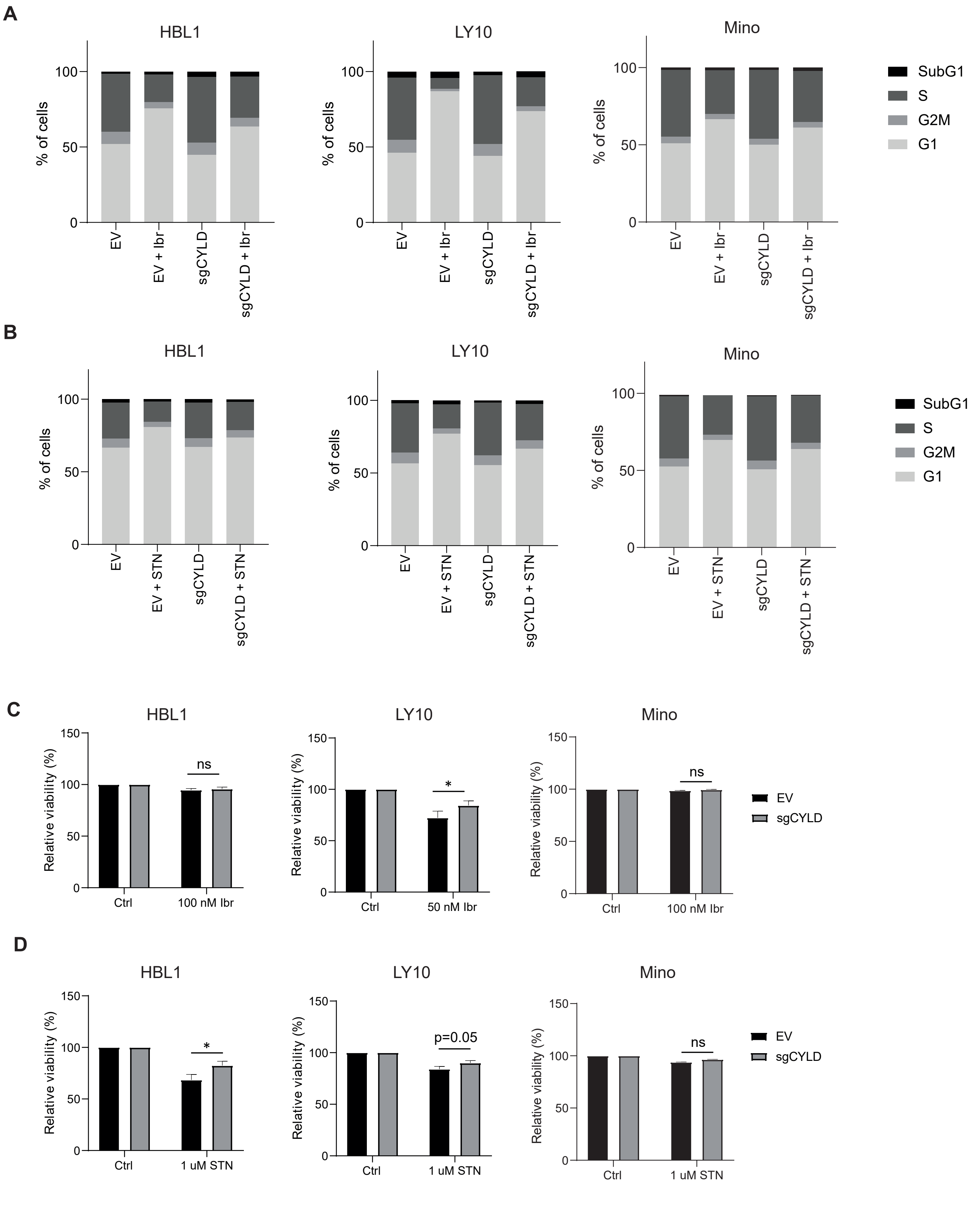

Supplementary Figure 6. (A-B) Flow cytometric analysis of the percentage of cells in Sub-G1 (BrdU-, <To-Pro-3-), G1 (BrdU-,To-Pro-3-), S (BrdU+) and G2/M (BrdU-, To-Pro-3+) after 48 hours of treatment with 50 nM Ibrutinib (A) or 500 nM Sotrastaurin (B) in HBL1, LY10 and Mino transduced with pLC-GFP-sgCYLD or empty vector control (EV). One representative experiment of three independent experiments is shown. (C-D) Viability assay in LY10, HBL1 and Mino transduced with lentiCRISPR-Cas9 without gRNA (empty vector; EV) or with sgCYLD treated for 3 days with indicated concentrations of Ibrutinib (C) or Sotrastaurin (D). The percentage of viable cells was defined as the percentage of cells being 7AAD negative. The mean ± S.E.M. of at least two independent experiments performed in triplicate is shown. $P>0.05$; ns (non-significant); $^*P<0.05$ using 2-way ANOVA with Bonferroni's multiple comparisons test.

Supplemental Figure 7

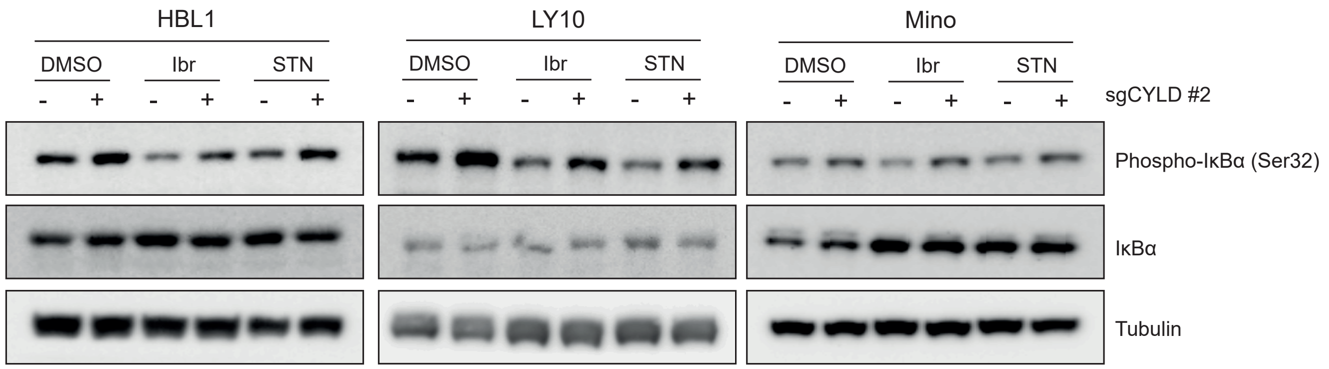

Supplementary Figure 7. Immunoblot analysis of (phosphorylated) IkBα in HBL1, LY10 and Mino transduced with lentiCRISPR-Cas9 (+/- sgCYLD) treated for 48 hours with 50 nM Ibrutinib or 500 nM Sotrastaurin as indicated. Cells were incubated with 5 µM proteasome inhibitor MG132 for 3 hours before harvesting. β-tubulin was used as loading control.
